# Supplementary material for: Are carbon emissions trading and green financial instruments synergistic? -Comprehensive quantitative research based on content analysis
Source: PLoS One. 2024 Mar 7;19(3):e0298601. doi: 10.1371/journal.pone.0298601 (PMC10919601; doi:10.1371/journal.pone.0298601)
Supplement: S1 Data — (ZIP) [file pone.0298601.s001.zip › Data/地级市Z_F工作报告.zip!/╔╜╬≈/╠1⁄2╘¡╩╨/└┤╫╘╣1⁄2╓┌║┼í╛┬φ┐╦╩2╛▌═°í┐_2013.docx]

各位代表：

　　现在，我代表市人民政府向大会报告工作，请予审议，并请市政协委员和其他列席人员提出意见。

　　一、2012年政府工作回顾

2012年，全市认真贯彻落实中央和省委、省政府的决策部署，把握稳中求进的总基调，全力以赴抓投资、稳增长、促转型、惠民生、保稳定，主要经济指标创近年来最好水平，许多事关全局的工作取得重大突破，社会文化等各项事业明显进步，在全省目标责任制考核中名列第一，率先转型跨越发展、建设一流省会城市迈出坚实步伐。

经济增速明显加快，主要指标位次前移。全年完成地区生产总值2	311.43亿元，增长10.5%；规模以上工业增加值782.96亿元，增长13.5%；固定资产投资1	320.63亿元，增长28.9%；社会消费品零售总额1	129.51亿元，增长16.1%；财政总收入454.49亿元，增长15.6%，公共财政预算收入215.67亿元，增长23.4%；城镇居民人均可支配收入22	587元，增长12.1%；农民人均纯收入10	079元，增长13.4%。

产业结构优化升级，发展方式加快转变。项目建设取得重大进展，江铃重汽等30个重大项目签约落地，太钢高性能碳纤维等25个高新技术项目开工建设，富士康苹果手机零组件等15个重大项目建成投产；现代物流、商务会展、城市综合体快速发展，中国（太原）煤炭交易中心正式运营，华润集团煤业总部落户我市。科技创新取得重大突破，企业创新能力持续提高，华顿实业和汾西重工建成国家级企业技术中心，中绿环保被确定为国家级国际科技合作基地；与高等院校、科研院所产学研合作迈出新步伐，组织实施50个自主创新项目，建设1个省级科技园、5个市级科技园；市技术转移促进中心被授予“中国创新驿站”，留学人员创业园成为国家级科技企业孵化器。武宿综合保税区获国家批准建立，对外开放迈出新步伐。

宜居城市建设大力推进，环境改善初见成效。新建、改建道路27条，综合改造小街巷45条，南客站广场及配套工程进展顺利，轨道交通建设规划获国务院批准，西山旅游防火通道及太古、太阳高速公路建成通车。积极创建国家卫生城市，深入开展城乡清洁工程达标活动和老旧片区整治，实现“两年大变样”的阶段性目标。全面改善省城环境质量初见成效，集中供热扩网2 785万平方米；太化、煤气化等8家重点污染企业关停；天然气置换工程全部完成；启动23个城中村整村拆除；“五河一渠”截污工程铺设管网46.21公里；市区煤炭消耗总量减少602万吨，采暖期燃煤总量下降35%。全年二级以上天数达到324天，比上年增加16天。全市绿化覆盖率、绿地率分别提高1.06、1.02个百分点，人均公园绿地增加0.57平方米。地下水位止降回升0.78米，集中式饮用水源地水质达标率稳定保持100%，荣获“全国节水型社会建设示范区”称号。

现代都市农业稳步发展，新农村建设有序推进。农业产业化龙头企业达到190个，实现销售收入101.13亿元。新建蔬菜标准园15个、设施蔬菜6	053亩，培育省级标准化养殖园9个。新增农民专业合作社445个，2万人脱贫。娄烦县马铃薯、小店区奶牛“一县一业”示范县和153个“一村一品”专业村建设顺利，区域主导产业逐步显现。农村新的“五个全覆盖”任务圆满完成，改造农村危房6	500户，新建、改建农村公路229公里，提高农村10万人饮水安全标准，85个新农村建设省级重点推进村、8个连片示范区建设有序推进。

民生改善成效显著，社会事业全面进步。“十件实事”全部完成。新开工建设保障性住房53	401套，竣工9	426套。新增公交车600台，建成主城区公共自行车服务系统，开通清徐、阳曲、高校园区等公交线路，我市入选首批国家创建“公交都市”示范城市。实施“百院兴医”工程，新建、改扩建25个项目。推进“百校兴学”工程，新建、改扩建59所项目学校、30所公办幼儿园。新增义务教育标准化学校122所，“联盟校”增加到152所。太原大学专科升本。城镇基本医疗保险转外就医范围扩大到全国。城乡低保标准进一步提高，四个主城区实现城乡低保标准一体化。城镇新增就业11.2万人，城镇登记失业率3.38%。加强和创新社会管理，大力推进社区管理网格化。推进平安省城创建活动，严厉打击各类违法犯罪，公众安全感不断提高。强化安全生产责任落实，组织开展安全隐患有奖举报，扎实开展“打非治违”专项行动。健全“源头治理、动态管理、应急处置”三道防线，信访总量下降42.1%。计生、外事、人防、对台、气象、防震减灾、档案、地方志、民族宗教、慈善、老龄、残疾人、妇女儿童等各项工作和事业都有新进步。

文化建设不断加强，文化强市再创佳绩。大力践行“三个核心价值观”。引深群众性文明创建活动，城市文明程度指数测评居省会、副省级提名城市第二。加强公民思想道德建设，树立“感动太原人物”等四类先进典型。组织“文化太原、幸福龙城”广场文化活动2 500余场。成功举办“三会一节一赛”。推进文艺精品塑造工程，编撰完成《太原通史》，晋剧《大红灯笼》、《傅山进京》等精品节目获国家奖励。认定第四批市级非物质文化遗产名录22项。高新区创意产业园等一批文化产业示范基地初步建成，文化产业增加值占到全市地区生产总值的6%左右。文化体制改革阶段性任务基本完成，再次荣获“全国文化体制改革工作先进地区”称号。

民主法制扎实推进，依法行政更加自觉。认真办理人大代表建议和政协提案，自觉接受人大、政协监督。提请市人大常委会制定修订《太原市终身教育促进条例》等5件地方性法规。积极支持人民政协履行职能，坚持重大事项向民主党派通报和协商制度。扎实推进“法治太原”建设，努力建设法治政府。大力推进以“两集中、两到位”为重点的审批流程再造，发展环境持续优化；深入开展“向人民汇报、请人民评议”和为人民群众“办实事、解难事”活动，干部作风明显转变。加大监察和审计力度，廉政建设取得新成效。国防后备力量进一步加强，实现全国双拥模范城“七连冠”。

这些成绩的取得，是省委、省政府和市委正确领导的结果，是市人大、市政协监督支持的结果，是全市人民团结奋斗的结果。在此，我代表市政府向全市人民，向驻并部队、武警官兵，向中央、省驻并单位，向各民主党派、工商联、人民团体、无党派人士，向各位人大代表、政协委员，向所有关心支持太原发展的海内外朋友们，表示崇高的敬意和衷心的感谢！

在肯定成绩的同时，也要清醒地认识到，我们的工作还有不少差距，发展还面临不少困难和问题。经济运行势头较好，但大项目、好项目还不够多，发展还不够快；结构调整有突破，但还不够协调，发展还不够平稳；生态环境改善初见成效，但面临形势依然严峻；工作推进力度较大，但发展还不够平衡；园区经济持续发展，但带动作用还不够明显；干部作风明显转变、发展环境持续优化，但还不够到位。这些问题事关全局，要采取有效措施，切实加以解决。

　　二、2013年政府工作主要任务

各位代表，今年是全面深入贯彻落实党的十八大精神的开局之年，是实施“十二五”规划承前启后的关键一年，也是全面推进转型综改试验先导区建设的重要一年。做好今年的政府工作，必须全面贯彻落实党的十八大精神和省、市第十次党代会部署，以邓小平理论、“三个代表”重要思想、科学发展观为指导，坚持主题主线，坚持稳中求进，以建设转型综改试验先导区为统领，以提高经济增长质量和效益为中心，深入推进工业新型化、农业现代化、市域城镇化、城乡生态化，全面加强经济、政治、文化、社会和生态文明建设，着力保障和改善民生，维护社会和谐稳定，解放思想、改革开放、凝聚力量、攻坚克难，为率先转型跨越发展、建设一流省会城市努力奋斗。

主要预期指标是：地区生产总值增长10%左右，规模以上工业增加值增长13%，固定资产投资增长25%，社会消费品零售总额增长15%，财政总收入和公共财政预算收入均增长12%，城镇居民人均可支配收入、农民人均纯收入分别增长11%和11%以上，城镇新增就业10.4万人，城镇登记失业率控制在4%以内，居民消费价格总水平涨幅控制在3.5%左右。

约束性指标是：万元地区生产总值综合能耗下降3.5%，六项污染物减排指标、万元地区生产总值二氧化碳排放量和万元工业增加值用水量均完成省下达任务。

围绕上述目标，在全面做好各项工作的同时，重点抓好以下七个方面的工作：

　　（一）强化创新驱动，加大开放招商，保持转型跨越发展的良好势头

在经济全球化时代，创新驱动深刻改变着一个城市和国家的命运。要把创新驱动作为核心战略，抓住新科技革命和第三次工业革命的机遇，大力发展创新型经济，加快一流新兴产业基地和自主创新基地建设。

要以建设太榆科技创新城为先导，推动企业与高等院校、科研院所产学研用深度融合，促进科技创新与新兴产业发展、传统支柱产业升级共生共长。加快发展太榆科技创新城核心区，集聚科研院所，加强招才引智，倾力打造科技孵化创新中心。增强企业在科技创新中的主体地位，支持和鼓励企业集团科研总部落户太原，创建科技成果交易平台，推进科研成果转化为现实生产力。充分发挥国家综改试验区的政策平台优势，深入推进工业振兴，重点发展装备制造、新材料、新能源、电子信息和食品加工等新兴支柱产业，改造提升传统产业，着力构建新型工业体系。加大对重点企业的服务力度，开工建设太钢硅钢冷连轧、太重高速铁路齿轮箱等30个重大项目，加快建设三一煤机、江铃重汽、中天信安防科技等重大项目，建成投产铁路货车修造基地、阳煤化工机械制造基地等20个重大项目。

要充分发挥省会城市资源和品牌优势，创新优惠政策，着力推进总部经济，加快发展高端服务业。在汾东新区、晋阳新区加快布局建设一批高端商务楼宇，重点引进跨国公司、国内百强企业在太原设立功能型总部，建设晋商、浙商、闽商、粤商、沪商等集团总部基地，加快汾酒、华润煤业等集团总部落地建设，培育具有国际影响力的大型企业集团和产业集群。积极发展现代服务业，培育壮大金融、现代物流、旅游会展、软件和信息技术服务业，建设罗克佳华物联网产业园。大力度引进国内一流商贸旗舰企业，整合优化汽车、建材、家具、农副产品、服装等批发零售服务业，优化功能布局，进一步提升省会城市区域中心的辐射力、带动力和影响力。

扩大招商引资是我市转型跨越发展的根本所在，必须以更开阔的胸怀、更开放的环境、更优惠的政策、更高效的服务，瞄准世界500强、中国500强、行业100强，瞄准央企和知名民企，抓好项目推介对接和跟踪服务，努力开创招大商、投大资、上大项目的新局面。要继续推进高新技术、经济开发、民营经济、综合保税、不锈钢等园区建设，北扩南展，扩大发展空间，提高承载能力，完善配套功能，优化发展环境，增强园区吸引和接纳国内外大集团、大企业的品牌效应。加强对国有企业的监督管理，增强企业经营发展能力。大力支持和帮助民营经济发展，切实解决民营企业发展中的土地、资金等困难，改善民营经济发展壮大的社会环境。

　　（二）强化基础设施建设，加快汾东新区和晋阳新区规划开发，建设现代宜居城市

认真落实国家建设太原城市群和省委、省政府构建“一核一圈三群”的战略部署，高标准规划建设管理省城，确立“以现代化为基干、特色化为神韵、人性化为根本”的理念，围绕塑造“唐风晋韵·锦绣龙城·清凉胜境”形象，按照建设新城、疏解老城、保护古城的思路，在加快现代化上求突破、在提高宜居度上下功夫、在增强便捷性上花气力，突出汾河概念、做好山水文章、打响清凉品牌，扎实推动城市南移、旅游西进，构建以汾河为轴线的多中心、组团式、生态型格局，形成山、水、城、林、人和谐共融，历史感、生态性、文化味并存的现代化都市气派，努力建设一流现代宜居城市。

要充分发挥规划引领作用。规划是城市发展的龙头，城市发展以规划论输赢，高水平规划是建设太原的根本前提。规划是城市第一资源、政府第一责任。各级政府必须以多留遗产、少留遗憾的历史眼光，宁受一时怨、不挨百年骂的责任担当，坚持规划的百年尺度和审美高度，树立和维护规划的权威性、原则性和严肃性。严格执行《关于进一步加强城乡规划管理的意见》，加大执法力度，严厉打击和控制违法违规建设。强化集约用地，控制总量、用好增量、盘活存量，推进土地利用方式转变。按照国际一流标准，进一步做好太榆科技创新城、汾东新区、晋阳新区、西山生态景观区、东山人文科技生态区、历史文化街区和城中村改造规划，完善城市快速交通、供热、防洪和污水收集处理体系，优化空间布局，提升规划建设水平。

要加快构建城市快速交通体系。增强区域性交通枢纽功能，推进高铁南客站和大西客专、太兴铁路建设，尽快形成全国性铁路网中心。启动轨道交通建设。开工建设东中环（太行路）、西中环（窊流路）、南中环、北中环，形成总长46公里的环状快速交通体系。建立网状干道体系，实现包括胜利街、府东街、南内环街、迎泽西大街、新建路、解放路、并州路、建设路、滨河东西路在内的城市主干道与中环、外环的全互通连接。改造滨河东西路部分节点，实现学府街、双塔街、亲贤街与滨河东路持续互通，启动滨河西路南延至清徐道路工程。对府东府西街、并州路进行改造，实现平面立体化，取消左转向位。逐步对迎泽大街、长风大街、南内环街进行平面立体化改造，畅通主干微循环。着力推进“公交都市”建设，完善快速公交系统，新增公交车600辆，建设公交专用道40公里；扩大公共自行车服务系统覆盖面，畅通自行车专用道路，提高服务水平；建设城市静态交通体系。完善龙城大街快速化体系，改造建设太茅路、长治路、西渠路、化章街、人民路等道路。高标准抓好政务大厅等9项城市功能配套工程建设，推进东西山生态绿化、晋阳湖改造、汾河生态景观等重点工程建设。严格落实《关于进一步规范城中村改造的若干意见》，加快拆迁安置房建设，抓好21个城中村整村拆除改造。大力推进小街巷综合整治、提档升级，完成60条小街巷改造，让人民群众实实在在感受到城市建设的成果。

要提升精细化管理水平。以创建国家卫生城市为契机，深入开展城乡清洁工程，加强重点部位的综合整治。加大“智慧城市”信息资源整合力度，从信息化入手，推进城市管理与国际先进水平对接，进一步加快“数字城市”向“智慧城市”升级转型。

　　（三）强化生态文明建设，着力综合治理，不断提升美丽太原建设管理水平

生态环境关系人民福祉，关系子孙后代和太原未来。要顺应人民群众对美好生活环境的期待，顺应一流省会城市的发展要求，以创建国家生态园林城市为抓手，大力加强生态文明建设和环境保护，以更高的标准、更严的要求，谋划推动省城环境质量全面改善，努力让太原天更蓝、水更清、地更绿，生活环境更美好。

优化发展空间。按照科学规划、合理布局的要求，继续清理关闭城市规划范围内选煤、储煤、煤焦发运、小火电、采石场等污染企业，逐步将散点布局的工业企业向园区集中，“腾笼换鸟”，造景增绿，提升城市建设水平，改善城市整体形象。

净化生态环境。大力推进集中供热、污水处理、垃圾处理、清洁燃料四个全覆盖。大幅减少燃煤总量，加快古交电厂三期等热源和供热管线建设，新增集中供热面积2 000万平方米。加快污水处理厂和管网建设，实现雨污分离，污水全收集、全处理，年内建成城南污水处理厂。开工建设具有国际水准的垃圾发电厂，加快餐厨垃圾、建筑垃圾的处理和综合循环利用，提高垃圾无害化处理率。加快气化太原步伐，实现民用天然气全覆盖。以治理雾霾天气为重点，开展PM2.5来源分析研究，整治扬尘和机动车排气污染。全面开展节能减排对标活动，狠抓重点领域、重点行业和重点企业节能工作。

绿化山川大地。以全省造林绿化现场会为契机，大力推进西山生态绿化和东山人文科技生态区工程，建设山川秀美的城市绿色长廊，推动全市造林绿化再上新台阶，彰显太原山水城市特色。开工建设汾河南延三期工程，加强汾河景区水质监管。加快晋阳湖生态景观工程建设，放大晋阳湖辐射带动效应，推动以晋阳湖为中心的新区建设。加强汾河水库等饮用水源地保护，全力保障饮水安全。

美化锦绣山河。城市建设和生态环境治理，都要寓审美于其中，实现城市园林化、生态化，创造绿色之美、人文之美、和谐之美、自然之美。

　　（四）强化社会建设，关注民生热点，让人民群众共享城市发展成果

让城市更美好，让人民更幸福，是政府一切工作的出发点和落脚点。民评民说是标准，民心民力是依靠，民意民声是准绳，民愿民盼是方向，民惠民富是目标。

要着力保障和改善民生。实施更加积极的就业政策，实现更高质量的就业。完善社会保障体系建设，提高社会保障标准和服务水平。完善保障性住房准入、退出、管理机制，新开工建设保障性住房35	000套，基本建成21	330套；对符合廉租住房保障条件的家庭逐步实现应保尽保；加快棚户区改造，尽快让棚户区居民住进清洁明亮、设施齐全的新居；加快征收住房安居工程建设，真正做到征收与安置同步，征收谁、改变谁、造福谁，在和谐征收、平安征收、公平征收中实现城市创新。规范市场秩序，保持物价稳定。完善食品药品安全监管机制，提高食品药品安全监管能力，让群众吃得更放心、用药更安全。

要加快社会事业发展。继续实施“百校兴学”工程，重点建设太原五中新校等标准化中小学和幼儿园；合理调整学校布局，推进新建住宅中小学、幼儿园配套建设；新增150所省级义务教育标准化学校；大力提高农村教育质量，鼓励支持规范民办教育，积极发展现代职业教育。继续实施“百院兴医”工程，启动市中心医院新建工程；深化医疗卫生体制改革，加强医疗救助和疾病控制，加快公共卫生体系和医疗卫生信息化建设。新建150个蔬菜直销店和100个平价商店，加快肉类蔬菜追溯体系建设，构建15分钟便民商圈。建设100个开放式篮球场，打造“汾河体育健身长廊”；办好太原国际马拉松、市第十届运动会暨第五届全民健身节等活动，做大做强体育产业。稳定低生育水平，提高人口素质，推进幸福家庭创建活动。加大社会救助力度，发展慈善事业和残疾人康复事业。认真做好第三次全国经济普查工作。

要坚持不懈抓好安全生产。坚决杜绝重大安全生产事故，努力不发生较大事故，大幅减少一般性事故，确保安全生产形势持续稳定好转。强化政府监管责任，全面实施挂牌责任制，完善安全生产责任测评体系。强化企业主体责任，加强安全生产基础建设，提高企业安全生产标准化水平。继续深入开展安全隐患有奖举报活动，形成政府、企业、社会共同推动安全生产的强大合力。加大“打非治违”力度，坚持安全执法“零容忍”理念，严格监督检查和责任追究，始终保持高压态势。深入开展消防和道路交通安全大检查，千方百计避免重特大事故发生。建立健全“统一管理、分级负责、政企结合、军民融合”的突发事件应急管理体制，加强应急队伍建设，切实提高应急救援能力。

要加强和创新社会管理。健全完善“源头治理、动态管理、应急处置”三道防线，深入推进社会管理创新综合试点工作。加强基层社会服务管理体系建设，提升管理水平和公共服务能力。多措并举，严厉打击各类违法犯罪。建立健全信访维稳和风险评估机制；认真落实领导接访、下访和包案制度，确保突发事件、疑难案件、交办案件及时有效解决；高度重视矛盾纠纷排查，对排查出的问题定期分析研判、有效化解，牢牢掌握工作主动权。

　　（五）强化文化建设，凸显龙城特色，打造传统与现代相结合的文化强市

文化是城市的灵魂，是城市赖以生存和发展的核心竞争力。文化承载着历史、传承着文明，以其强大的历史穿透力、文明渗透力、艺术感染力、民族凝聚力，发挥着人类精神之魂的独特作用。

太原作为历史文化名城，必须有历史文化遗存的实物载体。历史文化遗存是不可再生、不可替代的珍贵资源。要大力推进以崇善寺、纯阳宫、文庙、国民师范、文瀛湖为中心的历史街区保护，延续锦绣太原的城市文脉和历史记忆。建设以双塔寺为中心的大景区。推进晋祠大景区综合整治，启动明太原城保护复兴和晋阳古城大遗址保护工程，梦回龙城，再现历史辉煌。

太原作为闻名全国的传统工业城市，能源重化工基地已成为城市文化性格的重要组成部分。要利用煤气化公司、太化集团等工业遗址，发展文化创意产业，展示工业文明历史轨迹，创造工业文化特色旅游。

繁荣文化事业。巩固文化体制改革成果，增强公益性文化事业单位发展活力和转企改制院团的市场竞争力，打造一批精品力作。完善公共文化服务体系，推动城乡文化一体化发展。加快太原美术馆、博物馆布展工作，年内向公众开放。

加强精神文明建设。深入实施铸魂聚力工程，建设社会主义核心价值体系，大力践行“三个核心价值观”。引深公民道德建设工程，建设与一流省会城市相适应的思想道德体系。巩固文明创建成果，以创建全国文明城市为目标，深入开展城乡文明创建活动，进一步提高城乡居民文明素养。

　　（六）强化城乡统筹，建设都市特色农业，发展壮大县域经济

加大统筹城乡发展力度，推动市域城镇化与农业现代化同步发展，用工业化、城镇化带动农业现代化，促进城乡共同繁荣。

加快发展现代都市农业。以工业化理念谋划农业，以市场化、产业化、组织化的方式发展农业，集中打造一批各具特色、效益突出的现代都市农业产业园。深入推进新农村建设，围绕农民收入翻番，大力实施兴农富民十大工程。发展设施蔬菜2.5万亩。加快农村土地流转，引导适度规模经营，提升农业产业化水平，增强农业发展活力。突出城郊型农业特点，加快发展农家乐休闲旅游业。

发展壮大县域经济。按照扩权强县试点工作总体要求，着力在项目推进、环境建设、民生改善、社会管理创新等重点领域和体制机制上求突破。加快实施“大县城”战略，把县城作为城镇化发展的重要环节，引导生产要素、优势产业向县城集中，形成县域经济发展核心。按照功能划分，加大“一县一业”项目建设力度，培育发展主导产业，做到“一县一业”有主业、“一村一品”有品牌，努力实现县域经济快速增长。加强扶贫开发，加大对革命老区的扶持力度。

　　（七）强化职能转变，突出服务宗旨，创造转型跨越发展的综合优势

面对新形势、新目标、新任务和人民群众的新期待，要全面加强政府自身建设，转变政府职能，建设人民满意的服务型政府。

严格依法行政，建设法治政府。认真执行人大及其常委会的决议、决定，积极支持人民政协履行职能，自觉接受人大、政协和社会各界的监督，广泛听取各民主党派、工商联、无党派代表人士的意见和建议，提高人大代表建议、政协提案的办理质量和效率。完善科学民主决策机制，提高决策的科学化、民主化水平。进一步规范行政行为，严格依法行使权力、履行职责。加强监察和审计工作，坚决整治行政不作为、乱作为。

优化发展环境，建设效能政府。按照国务院行政审批制度改革精神、省委省政府和市委部署，继续推进政企分开、政事分开、政社分开。简政放权，进一步落实“两集中、两到位”，有所为有所不为，努力做到不该管的不管不干预，该管的切实管住管好。

改进工作作风，建设务实政府。空谈误国，实干兴邦。摆脱文山会海，力戒形式主义，深入基层调查研究。坚持对标一流、一事一表、例会调度、严格考评，创新工作方式，全力抓好落实。深入开展“访民生、知民情、解民事”走访活动，继续推进“向人民汇报、请人民评议”活动，主动接受群众监督，当好人民公仆。

保持清正廉明，建设廉洁政府。严格执行中央八项规定和省、市委有关要求，认真落实党风廉政建设责任制，着力加强制度建设，积极推进政务公开，强化对权力运行的监督制约。坚决反对铺张浪费，加强对“三公”经费支出的监督检查，加大对违法违规问题查处力度，做到干部清正、政府清廉、政治清明。

各位代表，新目标、新任务鼓舞着我们，新期盼、新愿景激励着我们。让我们紧密团结在以习近平同志为总书记的党中央周围，在省委、省政府和市委的坚强领导下，在市人大、市政协的监督支持下，紧紧依靠全市人民，夙夜在公、勤勉工作，抓住机遇、乘势而上，对标一流、奋力赶超，为我市率先转型跨越发展、建设一流省会城市努力奋斗！
